# Y Chromosome Material in Turner Syndrome

**DOI:** 10.7759/cureus.19977

**Published:** 2021-11-29

**Authors:** Abdullah Baris Akcan, Osman K Boduroğlu

**Affiliations:** 1 Department of Pediatrics, Division of Neonatology, Aydın Adnan Menderes University Faculty of Medicine, Aydın, TUR; 2 Department of Pediatrics, Division of Pediatric Genetics, Hacettepe University Faculty of Medicine, Ankara, TUR

**Keywords:** gonadoblastoma, mosaicism, y chromosome, fish, turner syndrome

## Abstract

Background

Turner Syndrome (TS) is a frequently identified chromosomal disease in humans characterized by short stature, sexual infantilism, streak gonads, primary amenorrhea, and a number of somatic anomalies. Approximately 55% of TS individuals have a nonmosaic 45,X karyotype. In addition, a cell line with a Y chromosome is present in 5% of patients, which is undetectable by the standard cytogenetic analysis. The identification of Y chromatin in some TS individuals has been associated with the development of gonadoblastoma. Therefore, it is important to exclude the presence of Y chromatin in TS individuals. In this study, it was planned to detect cases with mosaicism in terms of Y chromatin with the help of Y whole chromosome probes (WCP) from individuals with TS by fluorescence in situ hybridization (FISH) analysis.

Methodology

Forty-four patients with Turner syndrome, who were being followed up in the Genetics Unit, were contacted and invited for the study. Of the 44 invited patients, 28 responded to the invitation. In this study, it was planned to detect TS patients with mosaicism in terms of Y chromatin with the help of the Y whole chromosome probe.

Results

The majority of the cases (71.42%) included in the study carried pure X monosomy, which is the classical Turner syndrome karyotype. Other structural X chromosome aberrations, in isolated or mosaic forms, were less frequently represented. Y chromosome sequences were searched in 28 cases with Turner syndrome by the FISH method using Y whole chromosome probe. Y chromosome sequence was detected in one (3.5%) case of 28 cases.

Conclusions

It is recommended that individuals with Turner syndrome be screened for Y chromatin. Detection of this will provide information and guidance to individuals with Turner syndrome, especially in terms of the risk of developing gonadoblastoma, with advanced clinical consultation. This study was conducted to emphasize the importance of this.

## Introduction

The incidence of Turner syndrome (TS), first defined as a distinct clinical condition in 1938, is one in 4000 to 10000 live births or approximately one in every 2500 live female baby births [1-3]. Turner syndrome is one of the most common chromosomal abnormalities in females. Turner syndrome results when one normal X chromosome is present in a female's cells and the other sex chromosome is missing or structurally altered. The missing genetic material affects development before and after birth.

Turner syndrome is a syndrome characterized by short stature, gonadal dysgenesis, typical dysmorphic features, urinary, cardiovascular, and skeletal abnormalities, and a missing or structurally abnormal second sex chromosome [1-3]. This disorder is one of the most common human chromosomal abnormalities. The karyotypic anomaly in 40-60% of TS patients is monosomy 45,X. However, there are also anomalies such as mosaicism, Xp or Xq deletion, and isochromosome of the long arm of the X [4]. In addition, the Y chromosome is found in 5-6% of individuals with TS, with an additional 3% having a marker chromosome (a structurally abnormal chromosome that can not be detected by conventional cytogenetic methods) derived from the Y chromosome or another chromosome [5]. 

The Y chromosome is one of the smallest chromosomes in the human genome (approximately 60 megabases) and represents 2-3% of the haploid genome. Cytogenetic studies allow the detection of different Y regions. One or more gene GBY regions on the Y chromosome (gonadoblastoma locus on the Y chromosome) are considered to be the critical region involved in the development of gonadoblastoma from dysgenetic gonads [6]. 

Gonadoblastoma is the most common neoplasm of abnormal gonads and originates solely from the gonads. Gonadoblastoma cases are between 1-40 years of age, and their typical features are congenital abnormal gonads with a sexual development disorder. Observed gonadal anomalies are bilateral "streak" gonads, streak gonads on one side, and testis or indeterminate type of abnormal gonads on the other. The occurrence of gonadoblastoma in a person with a developmental disorder is dependent on the presence of a Y chromosome. A case with any gonadal dysgenesis and a Y chromosome is most likely to develop a germ cell tumour and, in particular, a gonadoblastoma. One-third of gonadoblastomas are bilateral, and they may be so small that they can merely be seen with a microscope. Malignant elements enlarge gonadoblastomas if they are not removed. Metastases of germinoma originating from within the gonadoblastoma are very rare, even if the tumour is large and bilateral [7]. 

Individuals with dysgenetic gonads and cells containing Y chromosome sequences have an increased risk of gonadoblastoma. The prognosis in patients with TS varies according to the karyotype. The risk of developing gonadoblastoma and virilization at puberty increases in individuals with Y chromosome sequences [7-9]. In individuals with TS, virilization of the external genitalia may indicate the presence of testicular tissue and presumably demonstrate the presence of Y chromosome sequences.

Individuals with TS should be investigated for Y chromosome mosaicism with an appropriate cytogenetic method, including fluorescence in situ hybridization (FISH). If the karyotype shows a marker chromosome of unknown origin, molecular studies using Y chromosome DNA probes may be useful. Moreover, if the patient has clitoromegaly or masculinized genitalia at birth and virilization at puberty, the possibility of Y chromosome mosaicism should be investigated [10]. If Y chromosome mosaicism is present, the risk of developing gonadoblastoma or dysgerminoma is 10-20% in the second decade of life [7-10]. The risk increases with increasing age, and the risk of developing gonadoblastoma in dysgenetic gonads increases up to 30% [9-11]. Hence, it is significant to demonstrate the presence of Y chromosome sequences in individuals with TS.

## Materials and methods

Forty-four patients with Turner syndrome, who were followed up in the Genetics Unit, were contacted and invited for the study by obtaining their addresses and phone numbers from the file records. Of the 44 invited patients, 28 responded to the invitation. This study was supposed to detect TS patients with mosaicism in terms of Y chromatin with the help of the Y whole chromosome probe (WCP).

Determination of Y chromosome sequences in patients with Turner syndrome by the FISH method

Individuals

Twenty-eight patients with Turner syndrome of various ages, diagnosed and followed up by the Genetics Unit, were evaluated by the FISH method to determine Y chromosome sequences. The age range of the patients was between 3 years 8 months and 30 years, and the mean age was 15 years (± 5.30 SD).

Method

For the FISH method, 10-20 ml of blood was taken from the antecubital vein from the patients who participated in the study, and Y chromosome sequences were searched with the help of standard methods, FISH method, and Y whole chromosome probes. Chromosome analyses of all patients were performed with the GTG banding method before FISH analysis. Patients who were shown to have Y chromosomes by the GTG banding method were not included in the study.

Cytogenetic Analysis

GTG Banding Method: The method used for chromosome analysis of the patients in the study group is summarized here. Peripheral blood samples were taken into tubes with sodium heparin, or injectors washed with 0.5 cc sodium heparin and kept at room temperature until seeding. The sample amount was 3-5 ml for children over 1 year in age and adults. In order to prevent microorganism contamination, the samples taken were cultured on the same day, and the samples that were kept at room temperature for more than 3 days were not preferred for culture.

The culture medium prepared for the blood samples in the amounts mentioned above was brought to room temperature for cultivation. An amount of 10 ml was placed in sterile culture tubes with patient names written on them under sterile conditions in the medium brought to room temperature. Following this, peripheral blood samples were added to the tubes containing the medium with the help of sterile green-tipped injectors (26-30 drops) (The concentration of white blood cells in each 10 ml culture medium should be 2 to 4X106cells per ml). After the blood addition process, the capped tubes were placed in the incubator at 37°C and kept in the incubator for 72 hours. (lymphocyte cells reach peak metaphase at 72 hours).

Harvesting Study

The tubes, which were removed from the incubator after 72 hours, were mixed gently, and 8 drops of colcemid were dropped into the tubes at room temperature. The mixed tubes were placed in an incubator at 37°C and kept in this incubator for 35 minutes. Tubes were removed from the incubator after 35 minutes and centrifuged at 2500 rpm for 10 minutes. After centrifugation, the supernatant in the tubes was discarded and 10 ml of potassium chloride (KCl) cooled to 37°C in vortex was added drop by drop to the tubes. Then tubes were placed back in the incubator. The tubes, which were kept in an incubator for 25 minutes, were then centrifuged again at 2500 rpm for 10 minutes. The supernatant in the tubes was discarded, and 10 ml of fixative was kept at -20°C in vortex was added drop by drop on the precipitate. The tubes, which were kept at 2-8°C for one night, were centrifuged for 10 minutes the next day, the supernatant was discarded, and 7 ml of fixative was added to the precipitate in a vortex. Afterwards, the supernatant was discarded in the centrifuged tubes once more, and 5 ml of fixative was added in a vortex. With the final centrifugation, the tubes were made ready for spreading. After the last fixative addition, the tubes that were ready to be spread on the preparations were centrifuged. The supernatant was discarded, and the precipitate remaining in the tubes was vortexed with a few drops of fixative. With the help of a pipette, 1-2 drops of material taken from the tube were spread on a dry and clean slide by dropping or spraying (the slides were preferably kept in methanol). The preparations were left to dry in a dry or humid medium, considering the environmental conditions. The dried preparations were then kept in a Pasteur oven at 60°C for one night. Thus, the chromosomes and cell nuclei were fixed on the slide. After this process, the slides were ready for staining.

An amount of 0.4/10 ml of trypsin was dissolved in distilled water and mixed with 50 ml of digestion buffer (DB). The prepared mixture was taken into a chalet. An amount of 0.2gr/100 ml methanol was mixed with 1 part of Leishman dye to 3 parts of Gurr Buffer. The prepared dye was used in the banding process. The aging process was completed, and the preparations were kept in the trypsin in the chalet for 13-14 seconds, then 2-3 ml of Leishman stain was poured on the preparations that were quickly washed with water. The preparations, which were treated with dye for 3 minutes, were then re-watered and allowed to dry. After checking the banding quality in the dried preparations with a light microscope, the preparations were placed in a xylene-filled chalet. A few drops of Entellan (Merck KGaA, Darmstadt, Germany) were dripped onto the xylene-treated preparations, and the preparations were sealed with a coverslip and adhered. After adhering, the preparations were ready for karyotyping.

The materials required for this process are briefly as follows for the peripheral blood culture medium: RPMI-1640 MEDIUM 100 ml (Sigma-Aldrich, Inc. Missouri, USA) storage temperature 2-8°C.; Fetal Bovine Serum 20 ml (SIGMA) storage temperature -20°C; Phytohmagglutinin 2 ml (Biochrom Ltd, Cambridge, UK) storage temperature -20°C; L-Glutamine 2 ml (Biochrom) storage temperature -20°C; Gentamicin 0.2 ml (Sigma) storage temperature 2-8°C; 10 ml Sterile Tube (Greiner Centrifuge Tubes, Sigma-Aldrich, Inc. Missouri, USA).

Harvesting Process Chemicals: Colcemid 8 drops (Irvine Scientific, Tilburg, The Netherlands) storage temperature 2-8°C; potassium chloride 10 ml storage temperature room temperature; fixative 3 parts methyl alcohol 1 part acetic acid (99.6%) (Merck, Darmstadt, Germany) storage temperature -20°C.

Banding Chemicals: Trypsin (Biochrom); Leishman Stain 0.2mg/100ml Methanol (Sigma) storage temperature room temperature; Gurr Buffer 1tb/1000ml distilled water (Gibco™ Gurr Buffer Tablets, Fisher Scientific Ltd, Loughborough, UK) storage temperature room temperature; DB Buffer 16 gr NaCl + 0.4gr KH2PO4 + 4.378g Na2HPO4/2000 ml distilled water; Xylene (Merck) storage temperature room temperature.

Fluorescent In Situ Hybridization (FISH) Method

A FISH study on chromosomes obtained from peripheral blood, bone marrow, and fluids such as amnion is a study based on hybridization and staining of chromosomes with some special fluorescent dyes. An overnight aging process is not applied to the preparations obtained as a result of culturing processes. After the spreading and drying stage, the preparations are ready for FISH.

Application: The preparations for FISH were stored at room temperature in an unlit medium. The preparations, which were kept under these conditions for one or a few days after spreading, were then subjected to FISH processes. FISH was done in a two-day period. On the morning of the first day, the hot water bath (bain-marie) was operated to ensure that the water temperature in the bain-marie was 72-73°C. The aging solution was placed in a 37°C incubator with a chalet, and its cap was closed. A moist bed was prepared by placing some moist gauze in the preparation box, and this bed was placed in an incubator at 37°C. After the denaturation solution, which was prepared freshly in a chalet in the afternoon, was kept in an incubator at 37°C for a while, it was placed in a bain-marie at 72-73°C and allowed to reach this temperature in the denaturation solution. During these procedures, the preparations to be FISHed were placed in the aging solution in the incubator and kept there for 30 minutes. The probe to be used was removed from -20°C and melted. A sufficient amount of probe (10 ml for each patient) was taken and placed in an Eppendorf tube so that no light could be seen in a partially dark environment. The stock probe was again raised to -20°C. The separated probe was wrapped with aluminum foil and ready for the denaturation process. The preparations extracted from the aging solution were passed through the alcohol series of 70%, 80%, and 95% at room temperature, which was previously prepared in the chalet, for 2 minutes. After the last alcohol permeation, the probe separated into the Eppendorf tube was placed in a bain-marie for denaturation, and the probe was kept at this temperature for 10 minutes. Preparations extracted from 95% alcohol were dried and placed in a denaturation solution at 72-73°C in a water bath. They were kept at this temperature for 3 minutes. After the denaturation process was completed, the preparations were quickly removed from the denaturation solution and passed through the 70%, 80%, and 95% alcohol series at -20°C for 2 minutes, respectively. The preparations from the cold alcohol series were dried, the probes kept in a water bath for 10 minutes were removed, and 10 ml probes were dripped onto the preparations with a micropipette in a partially light-free medium. The preparations were quickly covered with a lamel, and the edges of the lamel were adhered. After the adhering process, the preparations were placed on the moist bed, which was prepared beforehand and placed in the incubator, and it was removed in the incubator at 37°C. The preparations were incubated overnight. In the morning, the bain-marie was opened, and 40-50 ml of 0.5 sodium citrate solution (SSC)** **was placed in it with a chalet, and the temperature of the bain-marie was brought to 72-73°C. The preparations removed from the incubator were placed by removing the lamels to the bain-marie brought to this temperature. The preparations were then placed in a chalet containing 50 ml of 1X phosphate-buffered detergent (PBD) solutionat room temperature and held there for one minute. The dried preparations are ready for dyeing.

Staining: While the preparations were placed in a water bath in 0.5 SSC, DAPI (4′,6-diamidino-2-phenylindole) dye and antifaderemoved from -20°C were kept at room temperature for a while, and then two units of DAPI and eight units of antifade were prepared in a way to be 10 ml for each preparation in a partially dark medium. The prepared dye was wrapped in aluminum foil in an Eppendorf tube to prevent contact with light. The preparations extracted from 1X PBD were dried, and 10 ml of DAPI-antifade mixture was quickly dropped on the mand covered with a coverslip. The preparations that were ready for analysis were wrapped with aluminum foil to prevent their contact with light and were stored in the dark for analysis. The preparations were stored at -20°C to prevent loss of signals in other preparations in cases where the analysis process took a long time. The analysed preparations were stored in a dark medium at -20°C for later reuse.

FISH Chemicals: Probe 10 ml (Oncor, Gaithersburg, USA) storage temperature: -20°C; fluorescent dye DAPI + antifade 10 ml (ONCOR) storage temperature: -20°C; aging solution: 50 ml 2XSSC+0.5% NP-40 (ONCOR+SIGMA) storage temperature: room temperature; denaturation solution: formamide 25.5 ml (ONCOR) + 20XSSC 3.5 ml (ONCOR) + distilled water 7 ml used as fresh; firstst day washing solution: 70,80,95% ethyl alcohol 50 ml (Merck) storage temperature: room temperature +-20°C; second day washing solution: 1X PBD 50 ml (Oncor) storage temperature: 2-8°C.

FISH analysis for Y chromosome sequences in patients, metaphase chromosome preparations obtained by classical cytogenetic methods, "Total chromosome DNA Probe; Chromosome Y,Red" (CP5624-RW; ONCOR), as well as Y whole chromosome probes (CoatasomeTotal Chromosome Probes, Coatasome, Gaithersburg, USA) were used in accordance with the manufacturer's recommendations.

Statistics

Statistical analyses were performed with the help of a computer using SPSS (Statistical Package for Social Sciences) for Windows version 11.0 (SPSS, Chicago, USA). For numerical values, the result is expressed as mean±standard deviation and median, while for nominal values, it is expressed as percentage, and p<0.05 was considered to be significant.

Ethical issues

Approval from the ethics committee of Hacettepe University, dated 12.09.2003 and numbered LUT 03/24, was obtained before the study. This study followed the principles for human investigations outlined in the Second Declaration of Helsinki.

## Results

General characteristics of the study group

Twenty-eight cases with Turner syndrome followed in the Genetics Unit were included in the study. 

The age range of the patients was between 3 years 8 months and 30 years, and the mean age was 15 years (± 5.30 SD). When the disease was diagnosed, the ages of the patients ranged from 7 months to 26 years, with a mean age of 9.7 years (± 5.13 SD).

The main somatic anomalies and their incidence rates detected when the cases were diagnosed are given in Table 1.

**Table 1:** The main somatic anomalies and their incidence rates detected when the cases were diagnosed.

Diagnostic findings	Number	Percentage (%)
Short stature	24	85.71
Low hairline	18	64.28
Cubitus Valgus	18	64.28
Metacarpus stature	9	32.14
Webbed neck	9	32.14
Thorax anomaly	13	46.42
Increased nevus	5	17.85
Lymphedema	2	7.14

The distribution of 28 Turner syndrome cases participating in the study according to karyotype is shown in Table 2.

**Table 2:** Distribution of cases according to karyotype.

Karyoype	Number	Percentage (%)
45 X	20	71.42
46 X iso X	3	10.71
45 X / 46 X iso X	3	10.71
45 X / 46 XX	1	3.6
45 X ; 46 X,r(X)	1	3.6

The majority (71.42%) of the cases included in the study carried pure X monosomy, the classical Turner syndrome karyotype. Other structural X chromosome aberrations, in isolated or mosaic forms, were less frequently represented. Y chromosome sequences were searched by the FISH method in 28 cases with Turner syndrome using Y whole chromosome probe, and Y chromosome sequence was detected in one (3.5%) case out of 28 cases.

The karyotype of 28 cases, the number of metaphases analysed by the FISH method, and whether the Y chromosome sequence was detected are shown in Table 3.

**Table 3:** Study of cases with the Fluorescence In Situ Hybridization (FISH) method.

Case number	Karyotype	Metaphase number	FISH-Y Chromatin
1	45 X	25	_
2	45 X	22	_
3	45X/46XisoX	21	_
4	45X/46XisoX	20	_
5	45 X	24	_
6	45 X	25	_
7	45 X	20	_
8	45 X	20	_
9	45X/46XX	24	_
10	45 X	23	_
11	46 X iso X	21	_
12	45 X	21	_
13	45 X	20	_
14	45 X	22	_
15	46 X iso X	23	_
16	45 X	25	PositiveY(+)
17	45 X	20	_
18	45 X	20	_
19	45 X	24	_
20	46 X iso X	22	_
21	45 X	21	_
22	45 X/46 X iso X	20	_
23	45 X	23	_
24	45 X	21	_
25	45 X ;46 X,r(X)	25	_
26	45 X	25	_
27	45 X	24	_
28	45 X	21	_

**Figure 1:**
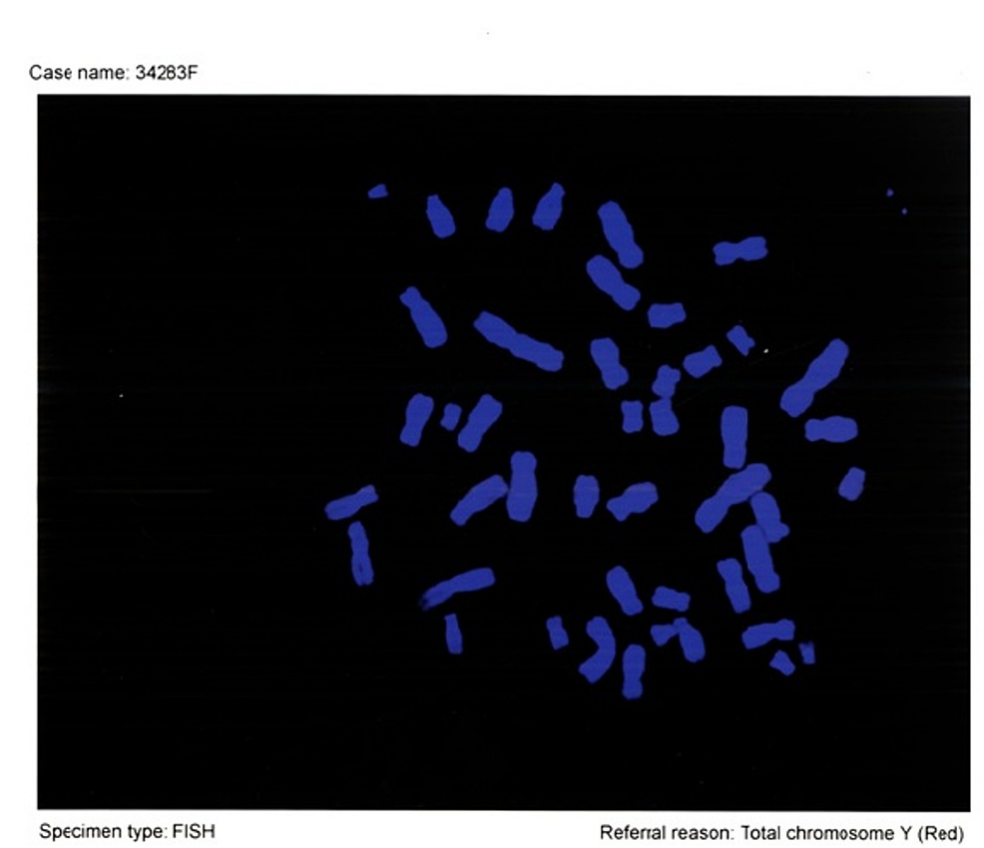
Case 1, screening for Y chromosome sequence by FISH analysis. In this case, the Y chromosome sequence could not be detected. fluorescence in situ hybridization (FISH)

In Figure 1, the screening of Case 1 for Y chromosome sequence by FISH analysis is shown. In this case, the Y chromosome sequence could not be detected.

**Figure 2:**
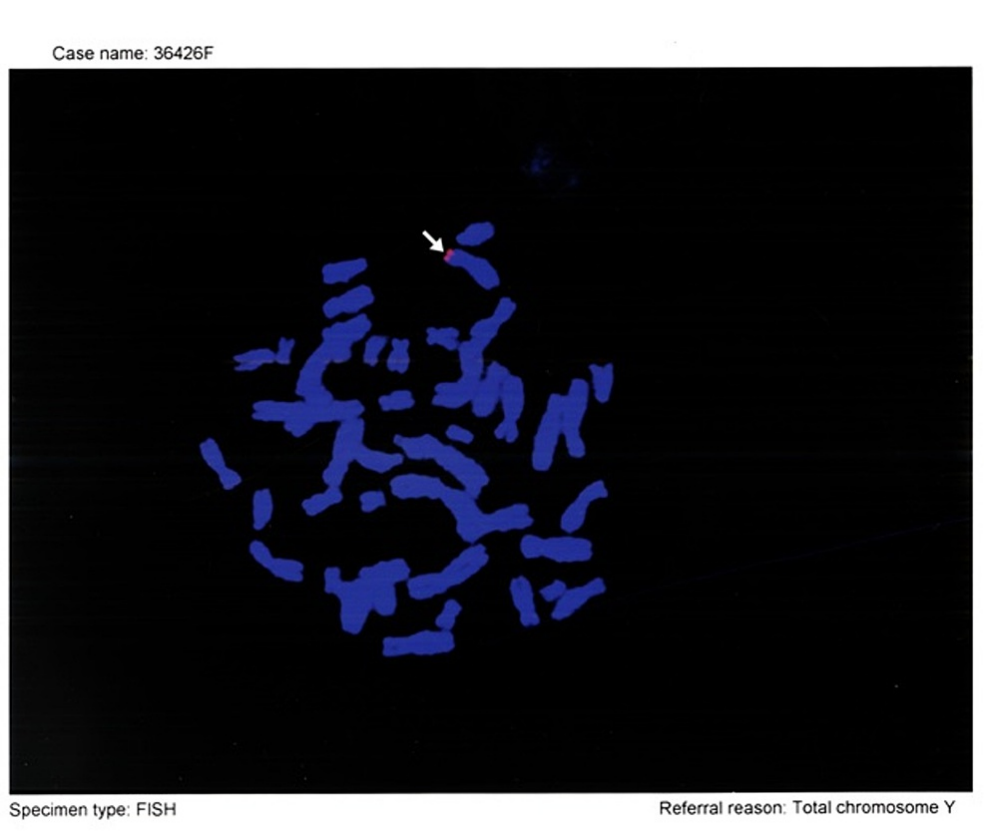
Case 16, screening for Y chromosome sequence by FISH analysis. The Y chromosome sequence was detected in this case (arrow). fluorescence in situ hybridization (FISH)

In Figure 2, the screening of Case 16 in terms of Y chromosome sequence by FISH analysis is shown. The Y chromosome sequence was detected in this case.

## Discussion

Various studies have been carried out in a large number of individuals with Turner syndrome with different karyotypes, and the rate of detecting the Y chromosome sequence varies between 6.9-60%, depending on the method applied [2,6,12]. The occurrence of gonadoblastoma in a person with a developmental disorder is dependent on the presence of a Y chromosome. A case with any gonadal dysgenesis and a Y chromosome is most likely to develop a germ cell tumor and in particular a gonadoblastoma. Individuals with dysgenetic gonads and cells containing Y chromosome sequences have an increased risk of gonadoblastoma.

The prognosis in patients with TS varies according to the karyotype type. The risk of developing gonadoblastoma and virilization at puberty increases in individuals with Y chromosome sequences [7-9]. Virilization of the external genitalia in individuals with TS may indicate the presence of testicular tissue and presumably indicate the presence of Y chromosome sequences. If TS patients have a Y-containing cell line, they have an increased risk for gonadal tumors. Therefore, TS patients are screened for Y chromosome and Y-specific sequences such as SRY, DYZ1, DYZ3, DYS132, ZFY, and TSP. Routine cytogenetic analysis does not always reveal cell lines, including the hidden Y chromosome. Therefore, further analysis may be required to assess neoplasm risks as part of the diagnostic process in TS cases. These complex techniques consist of polymerase chain reaction (PCR) and FISH analysis of peripheral blood and/or tissues such as gonads and buccal cells. Hidden Y chromosome mosaicism can be detected with this approach [6,13].

Gonadoblastoma can cause virilization at puberty. Its presence is usually accompanied by the presence of Y chromosome sequences. Current guidelines recommend testing for Y chromosome material only in TS patients with marker chromosomes and virilization to detect individuals at high risk of gonadoblastoma [14-15]. However, it has been suggested that cryptic Y chromosome material is a risk factor for gonadoblastoma in patients with TS. Therefore, it is argued that screening for cryptic Y chromosome material should be recommended in all patients with TS to detect individuals at high risk of gonadoblastoma [15].

In a publication made in 2020, it was discussed that prophylactic gonadectomy should be recommended in the diagnosis because of the increased tumor risk in patients with TS (TS + Y) with Y chromosome material [8]. In this study, spontaneous thelarche, spontaneous menarcherates, and the prevalence of gonadal tumors and malignancies were tried to be determined in TS patients with Y chromosome material and discussions about gonadectomy were included. In this multicenter cohort, 42% of girls with TS + Y had spontaneous puberty and 11% had spontaneous menarche supporting gonadal function. Tumor risk was similar to previous reports. To achieve informed decision-making, discussions about gonadectomy should include the potential for gonadal function and tumor risk, and they recommend that multidisciplinary teams provide up-to-date guidelines on gonadal management at diagnosis. Patients and their families should evaluate the factors that are most important and make an informed decision with the support of a multidisciplinary team.

In a publication of 2015, 60 TS patients were investigated in terms of Y chromosome and gonadoblastoma-Y-locus (GBY), and GBY locus was detected in seven patients and they were followed up. The presence of GBY locus in Turner patients who do not have Y chromosome signs in standard cytogenetic chromosome analysis can be revealed by sensitive molecular methods [16]. Binder et al. in 53 patients [17]; Coto et al. in 18 patients [18]; Fernandez et al. in 25 patients [19] found Y chromatin material at a rate of 3.3%, 26.6%, and 9.1%, respectively. In a study conducted by reviewing the literature in 2016, Y chromosome sequences and the prevalence of gonadoblastoma were evaluated using molecular techniques in TS patients. Based on these data, molecular analysis is indicated to detect Y-chromosome sequences in TS patients, regardless of their karyotype. The need for follow-up for gonadoblastoma was emphasized in patients who were positive for these sequences [20]. Canto et al. [21] investigated 107 individuals with 45,X karyotype in terms of Y chromosome sequences and found Y chromosome sequences in 10 patients (9.3%). They found gonadoblastoma in one patient and dysgerminoma in one patient who had under göne gonadectomy in six of these 10 patients (33%).

Twenty-eight patients with Turner syndrome at various ages who were diagnosed and followed up by Hacettepe University Genetics Unit were included in the study. The age range was between 3 years 8 months and 30 years, and the mean age was 15 years (± 5.30 SD). Y chromosome sequences were searched in 28 cases with Turner syndrome with the help of FISH method and Y whole chromosome probes. The results are shown in Table 3. Y chromosome sequence was detected in one (3.5%) case out of 28 cases (Figures 1, 2). Case 16 is shown to be screened for Y chromosome sequence by FISH analysis. Y chromosome sequence was detected in this case (Karyotype: 45 XO). Gonadectomy was recommended for the case and the patient was followed up with routine controls (Figure 2).

Individuals with TS should be investigated for Y chromosome mosaicism with an appropriate cytogenetic method, including FISH. If the karyotype shows a marker chromosome of unknown origin, molecular studies using Y chromosome DNA probes may be useful. In addition, if the patient has cliteromegaly or masculinized genitalia at birth and virilization at puberty, the possibility of Y chromosome mosaicism should be investigated [10]. Individuals with TS showing such features should be evaluated for the presence of Y chromosome sequences by advanced molecular cytogenetic and molecular genetic methods. One of the most important problems of clinical cytogenetics is to reveal the origin of chromosomal material that cannot be detected by the conventional method. Classification of this type of marker chromosome is important in revealing the phenotype-karyotype relationship and warning patients, especially those with the Y chromosome, about the increased risk of gonadal malignancy. The FISH method is an easy, reliable and effective method for detecting the marker chromosome.

## Conclusions

If Y chromosome mosaicism is present, the risk of developing gonadoblastoma or dysgerminoma is 10-20% in the second decade of life. The risk increases with increasing age, and the risk of developing gonadoblastoma in dysgenetic gonads increases up to 30%. Therefore, it is important to demonstrate the presence of Y chromosome sequences in individuals with TS. It is recommended that individuals with TS be screened for Y chromatin. Detection of this will provide information and guidance to the individual with TS, especially with regard to the risk of developing gonadoblastoma, with further clinical counselling.
